# Note on Intraperitoneal Injections

**Published:** 1897-03-20

**Authors:** Herbert E. Durham

**Affiliations:** Gull Research Student in Pathology, Assistant in the Throat Department, Guy's Hospital.


### NOTE ON INTRAPERITONEAL INJECTIONS.

By Herbert E. Durham, M.A., M.B.Camb., F.R.C.S. Gull Research Student in Pathology, Assistant in the Throat Department, Guy's Hospital.

Whilst intraperitoneal injections into experimental animals form an almost daily exercise in the laboratory, at present they are not commonly administered to man. A few words on technique may, therefore, not be superfluous. In the first place, most thorough cleanliness or asepsis must be observed; for cleansing, a compress of some antiseptic solution may be used, in order to soften the cuticular layers, followed by the use of the razor, whether hair be present or not. As regards the site of injection, experience with small animals teaches that the regions where tensely filled portions of the intestine (stomach, large intestine, cæcum) occur should be carefully avoided; and that by using a blunt needle damage to the small intestine can be uniformly and successfully avoided, even though the structure of its wall is so delicate. Since the blunt needle will not penetrate the skin, a small cut must be made with a knife or a tiny hole with a white hot cautery, which does not give rise to any discomfort. In operating upon man, therefore, it would be well to avoid the situation of the stomach transverse, colon, and sigmoid flexure, to make a small incision through the skin (if preferred with local anæsthesia), and to use a somewhat blunted needle. Experiments in the deadhouse have not been undertaken, as the conditions of the elasticity, &c., of the gut wall are so materially altered after death; but probably the risk of injuring the intestine is extremely slight or entirely negligeable.

Ten c.c. of a good sample of streptococcus serum would probably amply suffice for giving local peritoneal protection. The operator should be satisfied that the sample serum contains a sufficient quantity of an efficient antiseptic to ensure its sterility. Possibly it may be found that a rather larger quantity of fluid would be advisable, as by diluting the serum with an equal quantity of sterile peptone broth—the ordinary 'broth' of the bacteriological laboratory. Finally, it may be stated definitely that as far as the experimental evidence is concerned, the method of guarding against fatal contamination of the peritoneum in cases where some risk is entailed, is fully as convincing and uniform in results as was the case with antidiphtheritic serum before that substance was applied as a therapeutic agent to man.

